# The Relations between Druggists and Physicians

**Published:** 1881-05

**Authors:** 


### Editorial.

---

### THE RELATIONS BETWEEN DRUGGISTS AND PHYSICIANS.

In the preceding volume of this journal the subject of prescription filling was discussed and a decided opinion expressed.

Now, as then, we believe that the prescriber has the right to control the prescription as to re filling, provided his wishes, in this particular, be expressed plainly upon it, and no well appointed prescription house will attempt to thwart his intention. All this, however, does not secure the physician in the exclusive use of his prescription, unless apothecaries should be required to fill no prescription or order for medicine without the genuine signature of some physician, which is not, by any means, practicable. In the first place, it is not only the privilege, but the duty of prescriptionists to fill orders for mixtures or single articles of drugs or medicines (except poisons) without the name of a practitioner attached. Of course he must be allowed to sell an ounce of castor oil or a drachm of turpentine; and if so, why not mix them? So it will be perceived that the patient may copy the prescription and send to any drug store for the ingredients in his own name, and the doctor could not reasonably blame any one but his patient.

But there are other still more perplexing questions growing out of the sale of secret remedies by druggists.

Physicians are supposed to be opposed to such traffic and imposition upon the public, and they begin to feel that druggists whom they patronize and endorse should at least make some distinction between secret remedies and legitimate medicines. Hence there seems to be a disposition in the larger Northern cities to compromise on placing nostrums in the background—not to show them conspicuously. This we hope and believe is the first step toward a revolution on this subject. Pnysicians promise and profess not to "encourage the sale" of such remedies, and must feel that, to preserve consistency, they must do something which can be construed into a discouragement of such bold, unblushing offers of them for sale by their druggists.

It is perhaps impracticable to attempt the correction of the evil at once, particularly while there is rarely to be found a druggist who does not deal in nostrums. Moreover, it is said there exists sometimes private understanding, whether legitimate and reputable or not, between certain physicians and druggists, by which they are mutually benefitted, and which both parties feel it their interest to continue. The druggist seeks business for the phys cian, who in return secures the largest possible number of expensive prescriptions for his apothecary. Furthermore, it is hinted that occasionally a physician may be found who will accept a percentage on the prescriptions sent to a certain druggist as remuneration for his efforts in this direction By this arrangement the unscrupulous practitioner directly increases his income in proportion to the number of useless prescriptions with which he may feel inclined to tax his unsuspecting patient.

In view of these and perhaps other elements of union between certain members of the two professions, it would not be expected to find fidelity to medical ethics sufficient for such physicians to tear loose at once from these alliances. Hence, those having this subject at heart, and determined to lessen, at any rate, the traffic in nostrums, ask first that the trade be made less respectable by the course above mentioned—keeping t e articles out of public view.

This course, if p rsisted in, will final y separate, at least, the sale of legitimate medicines and secret humbug advertised preparations, and leave the latter unconnected in every way with true medicine. The drug store and the nostrum store will have no more connection than the dry goods store and blacksmith shop. Then, secret remedies having no longer any connection with legitimate medicines in the same house will gradually be lost sight of by the public as remdies for disease.

